# Selection and characterization of naturally occurring high acidification rate *Streptococcus thermophilus* strains

**DOI:** 10.1080/13102818.2014.966233

**Published:** 2014-10-21

**Authors:** Zoltan Urshev, Nadya Ninova-Nikolova, Daniela Ishlimova, Kalinka Pashova-Baltova, Michaela Michaylova, Tatyana Savova

**Affiliations:** ^a^ LB Bulgaricum Plc., Sofia, Bulgaria

**Keywords:** acidification, fermented milk, proteinase, *Streptococcus thermophilus*

## Abstract

Among *Streptococcus thermophilus* cultures, the principle component of yoghurt and cheese starters, a minority of strains forms the group of ‘H’-strains which show an unusually high acidification rate, grow faster and coagulate milk 3–5 hours earlier than the typical *S. thermophilus* cultures. A large-scale screening study was performed to select ‘H’-strains of *S. thermophilus* from more than 100 samples of home-made yoghurt, industrial yoghurt starters and single cultures, maintained in the LBB culture collection. Only four strains – LBB.TN1, LBB.M23, LBB.M34 and LBB.M60 – were isolated/selected due to their ability to form large yellowish colonies on milk agar, supplemented with beta-glycerophosphate and bromocresol purple. While in general *S. thermophilus* is described as a species with limited proteolytic capacity and in contrast to all other tested *S. thermophilus* cultures, the four selected strains invariably gave positive amplification product with the polymerase chain reaction when primers, specific for the membrane proteinase-coding gene *prt*S were used. The macrorestriction profiles of the genomic DNA of the four strains confirmed that they are non-isogenic and not related to each other. When grown in milk and compared to the control industrial strain LBB.A, the four strains showed a dramatically faster acidification, coagulating milk within four hours. The application of strain TN1 or M23 as adjunct culture to industrial yoghurt starter LBB.BY5-12 resulted in shortening the fermentation time with more than 30 min.

## Introduction

*Streptococcus thermophilus* is a key component of dairy starters. In yoghurt it is generally recognized that *Lactobacillus delbrueckii* ssp. *bulgaricus* has the leading role in providing milk protein-deriving amino acids and peptides necessary for bacterial growth, in that way supporting the proliferation of the weakly proteolytic *S. thermophilus* culture,[1] for which, otherwise, milk is not the optimal growth medium.[2] However, a minority of *S. thermophilus* strains, the so-called ‘H’-strains (form high acidification rate), have been found to grow rapidly in milk, always in correlation with the presence of the *prt*S proteinase in their membrane.[3,4]

Highly active starters, judged by their high acidification rate in milk, are welcome by dairy product manufacturers due to their economic feasibility. Therefore, the application of fast-acidifying *S. thermophilus* cultures in different starters or as adjunct cultures can greatly contribute to the competitiveness of bacterial starter preparations. There has already been an example of the successful introduction of a genomic region, containing the proteinase *prt*S gene, into *S. thermophilus* to convert it into a fast-acidifying culture.[5] The present study, however, deals with the results of a thorough screening process for the identification of naturally occurring *S. thermophilus* ‘H’-strains among cultures originating from home-made yoghurt, collected in Bulgaria, and preserved as pure cultures, potential industrial starters or simply yoghurt samples.

## Materials and methods

### Bacterial cultures and culture conditions

The screening for fast-acidifying *S. thermophilus* cultures covered eight industrial starters, 20 samples of home-made yoghurt collected in 1997 and 80 single-strain cultures collected in the period 1970–1997 and maintained in the LBB Culture Collection (LB Bulgaricum Plc., Sofia, Bulgaria). Ten-fold serial dilutions of each sample were directly plated on the surface of a differentiating milk–agar medium, supplemented with beta-glycerophosphate and bromocresol purple.[6] The plates were incubated for exactly 24 hours at 37 °C in an anaerobic atmosphere (Anaerocult A, Merck, Darmstadt, Germany). Fast-growing yellowish colonies were picked up and propagated in sterile 10% reconstituted skim milk powder or M17-broth [7] at 37 °C. The acidification rate and the proteolytic activity were determined for milk cultures, at an inoculation rate of 1%, while M17-broth cultures were used for DNA isolation. The selected ‘H’-strains LBB.TN1, LBB.M23, LBB.M34 and LBB.M60 were compared to a control *S. thermophilus* strain LBB.A, a component of industrial yoghurt starter LBB.BY5-12. Freeze-dried preparations of *S. thermophilus* TN1 or M23 (≥1 × 10^10^ CFU/g) were also used as adjunct cultures added in a 1:1 ratio to freeze-dried starter BY5-12 (*L. delbr*. ssp. *bulgaricus –* 5 × 10^8^ CFU/g; *S. thermophilus –* 1 × 10^10^ CFU/g) and their effect on the acidification rate was tested by inoculating 1 litre milk with 0.035 g of each powdered starter mix.

### Determination of acidification rate and proteolytic activity

The acidification rate of the cultures was determined in milk medium by measuring the active acidity (pH) during fermentation. The proteolytic activity was measured for 16 h cultures with the o-phthalaldehyde method of Church et al. [8] and presented as mmol/L methionine equivalents, calculated from the difference of fermented samples and non-inoculated milk and averaged for four parallel measurements.

### Primer design and PCR amplifications

The primer pair PRTSF (3′-TCGTCAGCGAATCAGCCTCAGA-5′) and PRTSR (3′-GCACGACGAGCAGCCTCGAT-5′) was designed with the PrimerBLAST tool [9] to amplify a 924 bp internal region within the *prt*S gene,[10] the sequence retrieved from GenBank Acc. No. FJ200299. Chromosomal DNA of all *S. thermophilus* cultures was obtained using the method of Slos et al. [11]. All amplifications were performed on a 9600 GeneAmp PCR System (Perkin-Elmer, Norwalk, Connecticut) in a 25 μL reaction mixture consisting of diluted VWR Taq DNA Polymerase Master Mix (VWR International, Haasrode, Belgium), 50 ng of template DNA and 10 pmol of each primer. The PCR programme was as follows: 1 cycle of 3 min at 95 °C; 30 cycles of 30 s at 93 °C, 30 s at 55 °C and 1 min at 72 °C; and 1 cycle of 7 min at 72 °C. The amplified products were separated by electrophoresis in a 2% agarose gel in TAE buffer at 100 V and visualized after staining with ethidium bromide. The size of the obtained product was determined using a suitable size marker (Gene Ruler 100 bp DNA Ladder, Thermo Scientific, Pittsburgh, PA, USA).

### Pulsed field gel electrophoresis

DNA for PFGE was prepared and digested with *Sma*I (Thermo Scientific) as described elsewhere.[12] The DNA fragments were electrophoretically separated in 1% PFGE-quality agarose gel on a CHEF-DRII PFGE unit (Bio-Rad, Hercules, CA, USA) in 0.5 × TBE buffer (45 mM Tris base, 45 mM boric acid, 1 mM EDTA, pH 8.3). The switch time ramp was set to 5–30 s for 20 h (block 1) and 30–50 s for 4 h (block 2) at 5 V cm^−1^. The Pulse Markers 0.1–200 kb and 50–1000 kb (Sigma-Aldrich, St. Louis, MO, USA) were used as size standards.

## Results and discussion

**Figure 1.**
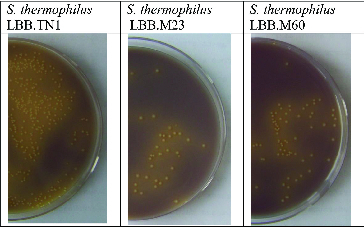
Fast-growing yellowish colonies formed by fast-acidifying *S. thermophilus* strains LBB.TN1, LBB.M23 and LBB.M60 on milk agar, supplemented with beta-glycerophosphate and bromocresol purple.[6]

Fast-acidifying *S. thermophilus* strains LBB.TN1, LBB.M23, LBB.M34 and LBB.M60 were initially identified among isolates that were able to form fast-growing yellowish colonies on the differential milk agar (Figure 1), where the majority of *S. thermophilus* cultures formed small white colonies unable to change the colour of the indicator dye from purple to yellow.

**Figure 2.**
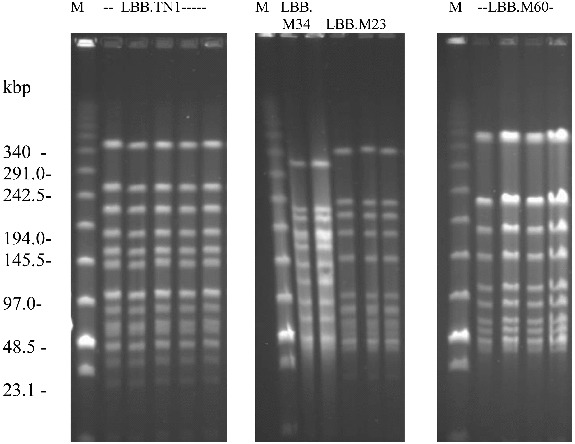
*Sma*I-macrorestriction profiles of fast-acidifying *S. thermophilus* isolates, corresponding to strains LBB.TN1, LBB.M34, LBB.M23 and LBB.M60. M-PFGE DNA size standard.

Fast-growing yellowish colonies were identified in 5 out of 20 home-made yoghurt samples. The comparison of the macrorestriction profiles of the respective isolates suggested the presence of only two distinguishable fast-acidifying strains. The first one, assigned as LBB.TN1, was common component in four home-made yoghurt samples, while all selected isolates in the fifth sample were identical and were assigned as LBB.M60 (Figure 2). Interestingly, the five yoghurt samples, containing strains LBB.TN1 or LBB.M60, also contained other strains of *S. thermophilus*, which, however, formed only small white colonies. The simultaneous presence of two or more strains of *S. thermophilus* in a single sample was found to be common in home-made yoghurt and has been described previously.[13]

Strain LBB.M34 was the only strain among 80 single cultures of *S. thermophilus* in the LBB culture collection that formed fast-growing colonies. Similarly only three isolates, originating from one of the 8 analysed industrial yoghurt starters, had the colony morphology of a fast-acidifying culture and showed identical macrorestriction profiles, consequently labelled *S. thermophilus* LBB.M23. *S. thermophilus* strains LBB.TN1, LBB.M23, LBB.M34 and LBB.M60 had dissimilar macrorestriction profiles to one another, suggesting that there was no relation between these cultures (Figure 2).

Differentiating milk-agar medium, supplemented with beta-glycerophosphate and bromocresol purple, was previously applied to select fast-acidifying *Lactococcus lactis* cultures.[6,14] The medium worked well for *S. thermophilus* cultures, provided that anaerobic conditions were used to obtain better differentiation of the colonies and incubation time was restricted to 24 hours to avoid erroneous readings.

**Figure 3.**
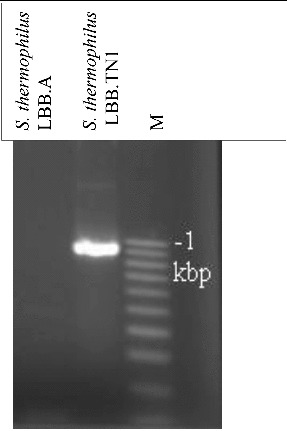
Amplification product obtained with primers specific for the membrane proteinase *prt*S gene in *S. thermophilus* strain LBB.TN1 against a negative control culture *S. thermophilus* LBB.A. M- 100 bp ladder DNA size marker.

**Figure 4.**
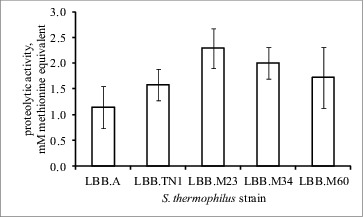
Comparison of the proteolytic activity of four fast-acidifying *S. thermophilus* strains LBB.TN1, LBB.M23, LBB.M34 and LBB.M60 and a control *S. thermophilus* strain LBB.A. Error bars represent the standard deviation (*n* = 4).

The PCR amplification of an internal region within the *prt*S gene yielded a product with the expected size in all four fast-acidifying *S. thermophilus* strains LBB.TN1, LBB.M23, LBB.M34 and LBB.M60, but not in the control strain LBB.A or in any other *S. thermophilus* culture from the LBB Culture Collection (Figure 3). This strongly suggested a link between the superior growth of strains LBB.TN1, LBB.M23, LBB.M34 and LBB.M60 on milk agar and the presence of a membrane proteinase gene. A direct comparison of the proteolytic activity of the four fast-acidifying strains and the control culture LBB.A showed accumulation of 50% more products of proteolysis in milk for strains LBB.TN1, LBB.M23, LBB.M34 and LBB.M60, a piece of indirect evidence for the presence of a membrane proteinase (Figure 4).

**Figure 5.**
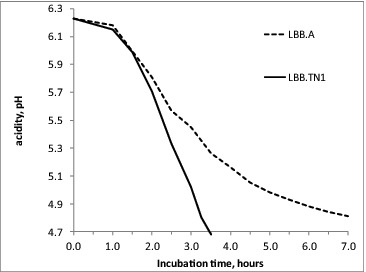
Typical acidification curve of fast-acidifying *S. thermophilus* strains, here represented by LBB.TN1, compared to a control industrial *S. thermophilus* strain LBB.A.

The relation between fast growth in milk and the presence of a membrane proteinase gene is already proven for *L. lactis*, where this important genetic determinant is located on a plasmid.[6,15] In *S. thermophilus*, the *prt*S gene is encoded in the chromosome, suggesting higher stability of the trait in this species. The comparison of the acidification curves of strains LBB.TN1, LBB.M23, LBB.M34 and LBB.M60, which were almost identical, with the same curve for the control *S. thermophilus* culture LBB.A, showed a dramatically higher acidification rate of the four selected strains (Figure 5). Clearly the higher proteolytic potential of the selected cultures contributed to increased acidification rate.

**Figure 6.**
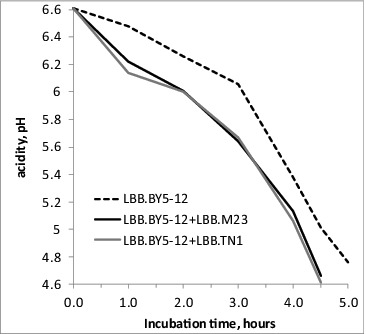
Acidification rate of freeze-dried yoghurt starter for direct application LBB.BY5-12, used in its original form or supplemented with an adjunct culture of fast-acidifying *S. thermophilus* strains LBB.M23 or LBB.TN1.

The effectiveness of three-component yoghurt starters, the third component being an additional *S. thermophilus* strain, conferring an additional property to the starter, such as improved texture or activity, was discussed in a previous work.[16] Here, the technological potential of the fast-acidifying strains was demonstrated when strains TN1 or M23 were applied as adjunct culture to industrial yoghurt starter LBB.BY5-12, to obtain a three-component starter. The fast-acidifying *S. thermophilus* strains contributed to a shortening of the fermentation time with more than 30 min when three-component starters were compared to LBB.BY5-12 (Figure 6). For this experiment, the three-component and control starters were applied in a freeze-dried form with direct inoculation of milk as required for direct vat starters (DVS). Compared to bulk starters, supplied in liquid or frozen form, the activity of DVS is negatively influenced by the lyophilization process, which makes the presence of fast-acidifying *S. thermophilus* strains even more valuable in the composition of freeze-dried starters for direct application.

## Conclusions

A combination of microbiological and molecular approach permitted the isolation/selection of four non-related fast-acidifying *S. thermophilus* strains, LBB.TN1, LBB.M23, LBB.M34 and LBB.M60, which possessed a membrane proteinase gene and showed a dramatically faster acidification rate compared to the control industrial strain *S. thermophilus* LBB.A. Although rarely found among *S. thermophilus* cultures, the fast-acidifying ‘H’-strains pay off for the selection efforts as they are promising candidates for the development of yoghurt and cheese starters with superior activity.
